# Prevalence and factors associated with hypertension among people living with HIV/AIDS on antiretroviral therapy in Uganda

**DOI:** 10.11604/pamj.2021.38.216.28034

**Published:** 2021-02-25

**Authors:** Gloria Lubega, Billy Mayanja, Joseph Lutaakome, Andrew Abaasa, Rebecca Thomson, Christina Lindan

**Affiliations:** 1 Medical Research Council/Uganda Virus Research Institute and London School of Hygiene and Tropical Medicine Uganda Research Unit, Entebbe, Uganda,; 2 London School of Hygiene and Tropical Medicine, London, United Kingdom,; 3 School of Medicine, University of California at San Francisco, San Francisco, United States of America

**Keywords:** Antiretroviral therapy, hypertension, prevalence, risk factors, body mass index, ART regimen

## Abstract

**Introduction:**

antiretroviral therapy (ART) has improved survival of People Living with HIV (PLWH); however, this has resulted in an increasingly high prevalence of non-communicable diseases (NCD) like hypertension. Hypertension is a major risk factor for cardiovascular and cerebral vascular disease, which are both associated with high morbidity and mortality rates. We studied the prevalence and factors associated with hypertension among PLWH on ART.

**Methods:**

we conducted a retrospective data analysis of PLWH on ART enrolled between 2011 and 2014 into a randomized double-blinded placebo-controlled trial investigating the safety of discontinuing cotrimoxazole prophylaxis (COSTOP) among PLWH in Central Uganda. We used the mean blood pressure (BP) measurements of the first four monthly clinic visits to define hypertension. Patients were categorised as: having normal BP (≤120/80mmHg), elevated BP (systolic >120-129, and diastolic ≤80), Stage 1 hypertension (systolic 130-139, or diastolic >80-89) and Stage 2 hypertension (systolic ≥140 or diastolic ≥90). Multiple logistic regression was used to evaluate factors associated with hypertension.

**Results:**

data from 2026 COSTOP trial study participants were analysed, 74.1% were women and 77.2% were aged 35 years and above. The overall prevalence of hypertension was 29%, of whom 19.5% had Stage 1 hypertension and 9.5% had Stage 2 hypertension. About 21.4% were overweight or obese. Factors independently associated with hypertension among PLWH on ART included increasing age (p≤0.001) and high body mass index (p≤0.001). Efavirenz (p≤0.001) and lopinavir/ritonavir (p=0.036) based regimen had lower odds of hypertension than Nevirapine based regimens.

**Conclusion:**

PLWH on ART have a high prevalence of hypertension, which rises with increasing age and body mass index (BMI) and among those on nevirapine-based ART. Implementation of hypertension prevention measures among PLWH on ART and integration of NCD and HIV care to improve patients’ management outcomes are required.

## Introduction

Sub-Saharan Africa (SSA) endures the highest burden of infectious diseases worldwide, including 70% of the global HIV burden [1]. Antiretroviral therapy (ART) has led to the reduction in new HIV infections, a decrease in HIV/AIDS-related deaths, and dramatic improvement in the survival and quality of life among people living with HIV (PLWH) [2]. However, PLWH are more at risk of non-communicable diseases (NCDs), particularly cardiovascular disease (CVD) including hypertension [2, 3]. In 2018, it was estimated that 1.4 million people were living with HIV in Uganda, and of these 73% were on ART [4]. Both HIV infection and ART play a role in the increased prevalence of CVD among PLWH [5].

HIV infection itself causes chronic inflammation even among those who are virally suppressed, and leads to early atherosclerosis [5, 6]. C-reactive protein, a marker of inflammation, has been shown to be high among PLWH [6]. Treatment with ART can cause lipodystrophy and metabolic syndrome, which also contributes to CVD [5, 6]. It has been proposed that dyslipidaemia may partly explain the higher prevalence of hypertension among PLWH on ART compared to those who are untreated. Specifically, use of protease inhibitors (PI) as part of an ART regimen have been associated with hyperlipidaemia [6, 7]. Many countries in SSA are experiencing an epidemiological transition, in which NCDs rather than infectious diseases are contributing more to the overall burden of disease [8]. CVDs are of particular concern because of the large number of people with HIV. We studied the prevalence of hypertension, and the association of ART and hypertension among PLWH on treatment in Uganda, who were enrolled in a trial to evaluate the effect of discontinuing of cotrimoxazole prophylaxis.

## Methods

**Study design:** we evaluated data collected as part of a randomized double-blind placebo-controlled non-inferiority trial evaluating the effects and safety of discontinuing cotrimoxazole prophylaxis among HIV-infected adults stabilized on ART (COSTOP). The trial investigated the difference in incidence of haematological and cotrimoxazole preventable events between those continuing on cotrimoxazole compared to those switched to a placebo. Enrolment and follow-up occurred from 2011 through 2014, and procedures have been described in detail elsewhere [9, 10].

**Study setting and participants:** COSTOP study participants were HIV-infected adults aged 18-59 years who were recruited from HIV care clinics in Masaka and Entebbe municipalities in Central Uganda, and evaluated at two research sites, one in each town. Eligibility into COSTOP study included having been on ART and cotrimoxazole prophylaxis for at least six months, being clinically asymptomatic, and having two prior CD4 cell counts of >250cells/mm^3^, including one within four weeks prior to enrolment. Those who had an acute illness, grade three or four anaemia, neutropenia or thrombocytopenia, known hypersensitivity to cotrimoxazole, and/or women who were in their first trimester of pregnancy were excluded. Eligible participants gave signed or thumb printed (for those who were illiterate) informed consent, prior to enrolment into the study.

Information on medical history and ART regimen were obtained by reviewing medical records; a physical examination was performed, and women of reproductive age underwent a urine pregnancy test. Participants provided information on sociodemographic characteristics by responding to an interviewer-administered questionnaire. Participants were asked to discontinue their regular cotrimoxazole and were then randomized to receive study-provided cotrimoxazole (960 mg) or a matching placebo. Participants were asked to return to the research clinic every month for the first three months following enrolment, and then every three months thereafter until the end of the study. Participants were followed for 12 to 36 months, depending on the date of enrolment.

**Measurements:** participants´ height was measured only at the enrolment visit. Weight and blood pressure (BP) were measured at each clinic visit. A digital calibrated electronic sphygmomanometer was used by a health worker to measure BP on the left arm with the participant seated upright in a chair, with legs uncrossed, after relaxing for a minimum of five minutes in the waiting area. Only one measurement was taken at each visit. For our analysis, the BP taken at the first 4 visits, including enrolment visit, were considered.

**Statistical analysis:** data analyses were performed using Stata 14 (Stata Corp, College Station, Texas 77845 USA) [11]. Participants with less than four blood pressure measurements during the first 4 clinic visits were excluded from the analysis. The WHO classification was used to categorise participants´ body mass index (BMI) in kg/m^2^ as being: underweight (<18.5 kg/m^2^), normal weight (18.5-24.9 kg/m^2^), overweight (25.0-29.9 kg/m^2^) or obese (>30 kg/m^2^) [12]. We classified ART regimens as: lopinavir/ritonavir (LPV/r), efavirenz (EFV) or nevirapine (NVP) based regimen.

The mean of the BP readings from the first four clinic visits (each one month apart) was calculated for our definition of hypertension. Using the 2017 American College of Cardiology (ACC) and American Heart Association (AHA) guidelines, we categorized participants as having: normal BP (≤120/80mmHg), elevated BP (systolic >120-129, and diastolic ≤80), Stage 1 hypertension (systolic 130-139, or diastolic >80-89), Stage 2 hypertension (systolic ≥140 or diastolic ≥90) [13]. We present frequency distributions of sociodemographic and medical characteristics, and show the proportion in each category who had Stage 1 and Stage 2 hypertension.

Our primary outcome variable was Stage 2 hypertension based on the above definition, or report of being on anti-hypertensive medications (regardless of measured BP). We used univariable and multivariable logistic regression to evaluate the association of participants characteristics with Stage 2 hypertension and present odds ratios (OR) and 95% confidence intervals (CI). All variables associated with hypertension at p-value<0.25 in bivariable analyses were included in an initial multivariable model and retained if they were still significantly associated at p≤0.05; age, sex and years on ART were retained to adjust for co-founding. We also performed a sensitivity analysis to evaluate the association of factors with having either Stage 1 or Stage 2 hypertension.

**Ethical considerations:** the parent COSTOP study was approved by the Uganda Virus Research Institute Research and Ethics Committee (UVRI REC), Uganda National Drug Authority, and Uganda National Council for Science and Technology. Additional approval for the secondary data analysis of hypertension described here was obtained from the UVRI REC and the London School of Hygiene and Tropical Medicine Ethics Committee. Participants were compensated for their time and received reimbursement for the cost of travel at each study visit.

## Results

**Baseline characteristics:** out of the 2180 enrolled COSTOP trial study participants, 2026 (93%) were included in this analysis while 154 (7%) participants with less than four BP measurements were excluded. The majority of included participants were women (74.1%) and most (77.2%) were aged 35 years and above, while 431 (21.3%) were overweight or obese. More than a half (58.3%) had been diagnosed with HIV for more than 5 years, but only 33.9% had been on ART for 5 years or more. The majority of participants (79.7%) were on a nevirapine based ART regimen, 16.7% on an efavirenz based regimen and 3.6% were on a lopinavir/ritonavir (LPV/r) based regimen (Table 1). There were 140 participants who were prescribed antihypertensive medications.

**Table 1:** baseline sociodemographic and clinical characteristics of 2026 HIV-infected Ugandan adults on ART who were evaluated for hypertension

Characteristic		Total		Normal/elevated BP		Stage 1 Hypertension		Stage 2 Hypertension	
		N=2026		N=1439(71%)		N=395(19.5%)		N=192(9.5%)	
		n	(%)	n	(%)	n	(%)	n	(%)
Gender									
	Female	1502	(74.1)	1084	(72.2)	271	(18.0)	147	(9.8)
	Male	524	(25.9)	355	(67.8)	124	(23.7)	45	(8.6)
Age, years									
	<35	462	(22.8)	392	(84.9)	56	(12.1)	14	(3.0)
	35-<45	880	(43.4)	629	(71.5)	184	(20.9)	67	(7.6)
	45-55	566	(27.9)	365	(64.5)	126	(22.3)	75	(13.3)
	>55	118	(5.8)	53	(44.9)	29	(24.6)	36	(30.5)
Body Mass Index (kg/m^2^)									
	Underweight (<18.5)	273	(13.5)	215	(78.8)	41	(15.0)	17	(6.2)
	Normal (18.5-24.9)	1,318	(65.2)	982	(74.5)	232	(17.6)	104	(7.9)
	Overweight (25.0-29.9)	341	(16.9)	202	(59.2)	88	(25.8)	51	(15.0)
	Obese ( ≥30 )	90	(4.5)	38	(42.2)	32	(35.6)	20	(22.2)
CD4 count prior to ART initiation, cells/mm^3^									
	<250	1,698	(89.9)	1220	(71.9)	313	(18.4)	165	(9.7)
	250-350	156	(8.3)	105	(67.3)	38	(24.4)	13	(8.3)
	>350	34	(1.8)	21	(61.8)	10	(29.4)	3	(8.8)
Duration of HIV infection, years									
	<5	837	(41.7)	613	(73.2)	154	(18.4)	70	(8.4)
	≥ 5	1,171	(58.3)	810	(69.2)	240	(20.5)	121	(10.3)
Duration on ART, years									
	< 5	1,334	(66.1)	960	(72.0)	254	(19.0)	120	(9.0)
	≥ 5	684	(33.9)	471	(68.9)	141	(20.6)	72	(10.5)
Current ART regimen									
	NVP-based	1571	(79.7)	1075	(68.4)	327	(20.8)	169	(10.8)
	EFV-based	329	(16.7)	264	(80.2)	48	(14.6)	17	(5.2)
	LPV/r-based	71	(3.6)	55	(77.5)	14	(19.2)	2	(2.8)

ART = antiretroviral therapy; EFV = Efavirenz; LPV/r = lopinavir/ritonavir; NVP = nevirapine; BP = Blood Pressure

**Prevalence of hypertension:** 395 (19.5%) participants had Stage 1 hypertension and 192 (9.5%) had Stage 2 hypertension. There were slightly more men (32.3%) with either Stage 1 or Stage 2 hypertension than women (27.8%). The proportion with either Stage 1 or Stage 2 hypertension increased with age. Nearly a third (30.5%) of those aged >55 years or more had Stage 2 hypertension, and about half of all participants in that age group (55.1%) had either Stage 1 or Stage 2 hypertension. The proportion who met either definition of hypertension, also increased with increasing BMI; with 57.8% of those who were obese having either Stage 1 or Stage 2 hypertension. Participants taking an NVP-based regimen were more likely to have Stage 2 hypertension (10.8%), than those taking EFV (5.2%) or LPV/r (2.8%). The proportion of participants with Stage 1 hypertension was similar among those taking an NVP-based regimen (20.8%) and those taking an LPV/r-based regimen (19.2%) (Table 1).

**Factors associated with hypertension:** we evaluated the factors associated with Stage 2 hypertension and found that increasing age was strongly associated with hypertension. Participants who were aged over 55 years old had 17.6 (CI 8.8-35.1) greater odds of having hypertension compared to those who were aged 35 years old or less. The odds of having hypertension also increased with increasing BMI. Participants on an EFV-based regimen (aOR 0.41, CI 024-0.70) or on LPV/r-based regimen (0.21, CI 0.05-0.90) were less likely to have hypertension than those on an NVP-based regimen. Gender, duration with an HIV diagnosis, duration on ART, or CD4 cell count at ART initiation were not found to be associated with hypertension (Table 2).

**Table 2:** association of sociodemographic and clinical characteristics with Stage 2 hypertension among 2026 HIV-infected Ugandan adults

Characteristic		Unadjusted			Adjusted		
		OR	(95% CI)	p-value	AOR	95% (CI)	p-value
**Gender**							
	Female	1			1		
	Male	0.87	(0.61-1.23)	0.42	0.93	(0.63-1.38)	0.735
**Age, years**							
	<35	1			1		
	35-<45	2.64	(1.46-4.76)	0.008	2.67	(1.47-4.85)	0.001
	45-55	4.89	(2.70-8.86)	<0.001	5.31	(2.91-9.69)	<0.001
	>55	14.05	(6.84-28.85)	<0.001	17.61	(8.84-35.08)	<0.001
**Body Mass Index, kg/m^2^**							
	Underweight (<18.5)	0.78	(0.46-1.32)	0.345	0.68	(0.38-1.22)	0.194
	Normal (18.5-24.9)	1			1		
	Overweight (25.0-29.9)	2.05	(1.43-2.94)	0.001	2.1	(1.43-3.11)	<0.001
	Obese ( ≥30)	3.34	(1.94-5.72)	<0.001	3.73	(2.10-6.62)	<0.001
**CD4 count prior to ART initiation, cells/mm^3^**							
	<250	1					
	250-350	0.84	(0.47-1.52)	0.574			
	>350	0.9	(0.27-2.97)	0.862			
**Duration of HIV infection, years**							
	<5	1					
	≥5	1.26	(0.93-1.72)	0.138			
**Duration of ART therapy, years**							
	<5	1			1		
	≥5	1.19	(0.87-1.62)	0.267	1.1	(0.79-1.54)	0.562
**Current ART**							
	NVP-based	1			1		
	EFV-based	0.45	(0.27-0.76)	0.002	0.41	(0.24-0.70)	0.001
	LPV/r-based	0.24	(0.06-0.99)	0.032	0.21	(0.05-0.90)	0.036

ART = antiretroviral therapy; EFV = Efavirenz; LPV/r = lopinavir/ritonavir; NVP = nevirapine

**Results of sensitivity analysis:** we performed a sensitivity analysis to evaluate factors associated with a combined outcome of having either Stage 1 or Stage 2 hypertension. Findings were similar, except that men had greater odds of having either stage of hypertension than women (aOR 1.5, CI 1.17-1.93), and those who were underweight were less likely to have hypertension (aOR 0.67, CI 0.47-0.96).

## Discussion

In this study of adults on ART in central Uganda, we found that nearly 30% of participants had hypertension based on combining Stage 1 and Stage 2 hypertension according to the new ACC and AHA guidelines. We also found that hypertension was associated with increasing age, increasing body mass index (BMI) and being on a nevirapine-based ART regimen.

The prevalence of hypertension has been reported to vary widely, from 8% among HIV infected adults in rural Uganda [14], to 70% among those who were older [15-17]. A pooled estimate of elevated BP among those greater than 50 years of age from 15 countries in SSA was found to be 57% [18], and another meta-analysis among HIV uninfected adults in the region found an overall prevalence of hypertension of 16.2% [19]. We used the recent ACC and AHA guidelines that use lower blood pressure cut offs for hypertension definition because those previously considered to have ‘pre-hypertension´ were also found to have a high risk of cardiovascular disease [13, 20]. These guidelines allow for earlier preventive interventions like changes in lifestyle and increased exercise so as to lower the BMI without necessarily starting medication. Therefore, comparison with other studies from SSA need to take into consideration the cut-offs for hypertension and elevated BP that have been used. A systematic review and meta-analysis done by Nduka *et al*. reported a higher prevalence of hypertension among participants on ART compared to those who were ART naïve [21]. While previous studies included participants initiating ART, our study included PLWH who were already stable on ART and this might explain the high prevalence of hypertension. We found a strong association between hypertension and increasing age, with prevalence of hypertension among those aged above 55 years being almost 18 times that among those aged 35 years and below. Other studies have reported similar findings among PLWH [22], as well as among HIV uninfected adults [14, 23]. Aging is associated with a permanent loss of elastin, deposition of collagen and protein glycation that occurs in the vascular cell walls. This leads to arteriosclerosis (stiffening and ageing of the arteries), which increases both systolic and pulse pressure in the elderly [24].

The association between hypertension and increasing body mass index (BMI) has previously been reported [14, 22, 23]. The risk of hypertension is reported to increase by 49% for every five unit increase in BMI [25]. BMI is an important modifiable risk factor, yet, improving healthy lifestyle choices to reduce BMI has not been given priority in reduction of hypertension in developing countries [26]. Prevention programs focusing on weight reduction and living healthy lifestyle through exercise and eating healthy are required to reduce overweight and obesity among PLWH. We found that Nevirapine-based ART regimens were associated with higher odds of hypertension, a finding that is supported by previous studies [7, 22]. Contrarily, Crane *et al*. reported a higher risk with ritonavir boosted lopinavir based regimen compared to other regimens [27]. A systematic review and meta-analysis of studies done among HIV infected patients reported a significantly increased risk of hypertension associated with ART exposure [21]. Some previous studies have reported increased lipid levels most especially with PI-based regimens and this has been proposed as one of the mechanisms through which ART leads to hypertension [6, 28]. Similar to our study findings, some other studies have also reported a lower risk of hypertension with a PI regimen [7, 28]. More research is therefore required to elucidate the mechanism through which ART leads to hypertension.

In our sensitivity analysis, using the ACC and AHA guidelines to define hypertension as Stage 1 and 2, similar factors were found to be associated with hypertension except, the male gender that became significantly associated with hypertension and while the association between ritonavir boosted lopinavir and hypertension was not significant. Hypertension is a major risk factor for cardiovascular and cerebrovascular diseases and chronic kidney failure [15]. There is pressing need for prevention and proper management of hypertension among PLWH to prevent adverse complications of hypertension and improve their health. This could be improved through integration of HIV and hypertension care services.

**Strengths and limitations of the study:** the strengths of our study were that we studied PLWH who were stable on long term ART attending an ambulatory clinic and the use of the ACC and AHA guidelines. However our study had limitations, since it was a secondary data analysis, we could not analyse for some important factors in the relationship between HIV and hypertension that were not collected in the main COSTOP trial like; alcohol use, smoking and renal disease.

## Conclusion

The high prevalence of hypertension found among PLWH on ART in our study, and the identified associated factors need interventions to mitigate the consequences of hypertension like cardiovascular and cerebral diseases. Whereas we can´t do much about older age that was associated with hypertension, something can be done about the other associated modifiable factors. It is relieving that the current ART policy recommends using non nevirapine-based regimens as we found that nevirapine-based ART regimens were associated with hypertension. Body mass index is a modifiable risk factor and prevention programs targeting weight reduction and healthy eating in addition to regular physical exercises should be enhanced among all the patients. Integration of hypertension and HIV management should be promoted for optimal access and adherence to both ART and antihypertension medications and better management outcomes. More operational research is required to establish the best models of integrating hypertension and HIV care as well as implementation of hypertension prevention measures among PLWH on ART.

### What is known about this topic

- People living with HIV have an increased risk of hypertension;
- Hypertension is a major risk factor for cardiovascular and cerebral vascular disease, which are both associated with high morbidity and mortality rates;
- Both HIV and ART may play a role in the increased risk of hypertension among PLWH.

### What this study adds

- We found a high prevalence of HIV among PLWH in Uganda;
- Modifiable risk factors found to be associated with hypertension among PLWH in Uganda were body mass index, Nevirapine based regimen. Prevention of these factors reduces hypertension among PLWH;
- Increased need for improved management of hypertension among PLWH.

## References

[1] World Health Organization (WHO) Health Statistics and Information Systems.

[2] Bain LE, Gwain GC (2019). Cardiovascular Disease and HIV Infection in sub-Saharan Africa: Misplaced Priorities in the Public Health and Research Agendas?. Front Cardiovasc Med.

[3] Xu Y, Chen X, Wang K (2017). Global prevalence of hypertension among people living with HIV: a systematic review and meta-analysis. J Am Soc Hypertens.

[4] UNAIDS Uganda factsheets.

[5] Cerrato E, Calcagno A, D'Ascenzo F, Biondi-Zoccai G, Mancone M, Grosso Marra W (2015). Cardiovascular disease in HIV patients: from bench to bedside and backwards. Open Heart.

[6] Dau B, Holodniy M (2008). The Relationship Between HIV Infection and Cardiovascular Disease. Curr Cardiol Rev.

[7] Shaffer D, Hughes MD, Sawe F, Bao Y, Moses A, Hogg E (2014). Cardiovascular disease risk factors in HIV-infected women after initiation of lopinavir/ritonavir-and nevirapine-based antiretroviral therapy in Sub-Saharan Africa: A5208 (OCTANE). J Acquir Immune Defic Syndr.

[8] Atiim GA, Elliott SJ (2016). The Global Epidemiologic Transition: Noncommunicable Diseases and Emerging Health Risk of Allergic Disease in Sub-Saharan Africa. Health Educ Behav.

[9] Anywaine Z, Abaasa A, Levin J, Kasirye R, Kamali A, Grosskurth H (2015). Safety of discontinuing cotrimoxazole prophylaxis among HIV infected adults on anti-retroviral therapy in Uganda (COSTOP trial): Design. Contemp Clin Trials.

[10] Anywaine Z, Levin J, Kasirye R, Lutaakome JK, Abaasa A, Nunn A (2018). Discontinuing cotrimoxazole preventive therapy in HIV-infected adults who are stable on antiretroviral treatment in Uganda (COSTOP): A randomised placebo controlled trial. PLoS One.

[11] StataCorp (2015). Stata Statistical Software: Release 14.

[12] World Health Organization (WHO) Global Database on Body Mass Index: an interactive surveillance tool for monitoring nutrition transition.

[13] Whelton PK, Williams B (2018). The 2018 European Society of Cardiology/European Society of Hypertension and 2017 American College of Cardiology/American Heart Association Blood Pressure Guidelines: More Similar Than Different. JAMA.

[14] Sander LD, Newell K, Ssebbowa P, Serwadda D, Quinn TC, Gray RH (2015). Hypertension, cardiovascular risk factors and antihypertensive medication utilisation among HIV-infected individuals in Rakai, Uganda. Trop Med Int Health.

[15] Ataklte F, Erqou S, Kaptoge S, Taye B, Echouffo-Tcheugui JB, Kengne AP (2015). Burden of undiagnosed hypertension in sub-saharan Africa: a systematic review and meta-analysis. Hypertension.

[16] Naidoo VA, Martinson NA, Moodley P, Joyimbana W, Mothlaoleng K, Abraham P (2020). HIV Prevalence and Morbidity in Older Inpatients in a High HIV Prevalence Setting. AIDS Res Hum Retroviruses.

[17] Bosu WK, Reilly ST, Aheto JMK, Zucchelli E (2019). Hypertension in older adults in Africa: a systematic review and meta-analysis. PLoS One.

[18] Kaze AD, Schutte AE, Erqou S, Kengne AP, Echouffo-Tcheugui JB (2017). Prevalence of hypertension in older people in Africa: a systematic review and meta-analysis. J Hypertens.

[19] Twagirumukiza M, De Bacquer D, Kips JG, de Backer G, Stichele RV, Van Bortel LM (2011). Current and projected prevalence of arterial hypertension in sub-Saharan Africa by sex, age and habitat: an estimate from population studies. J Hypertens.

[20] Guo X, Zhang X, Guo L, Li Z, Zheng L, Yu S (2013). Association between pre-hypertension and cardiovascular outcomes: a systematic review and meta-analysis of prospective studies. Curr Hypertens Rep.

[21] Nduka CU, Stranges S, Sarki AM, Kimani PK, Uthman OA (2016). Evidence of increased blood pressure and hypertension risk among people living with HIV on antiretroviral therapy: a systematic review with meta-analysis. J Hum Hypertens.

[22] Brennan AT, Jamieson L, Crowther NJ, Fox MP, George JA, Berry KM (2018). Prevalence, incidence, predictors, treatment, and control of hypertension among HIV-positive adults on antiretroviral treatment in public sector treatment programs in South Africa. PLoS One.

[23] Bosu WK, Aheto JMK, Zucchelli E, Reilly ST (2019). Determinants of systemic hypertension in older adults in Africa: a systematic review. BMC Cardiovasc Disord.

[24] Williams B (2016). Vascular ageing and interventions: lessons and learnings. Ther Adv Cardiovasc Dis.

[25] Jayedi A, Rashidy-Pour A, Khorshidi M, Shab-Bidar S (2018). Body mass index, abdominal adiposity, weight gain and risk of developing hypertension: a systematic review and dose-response meta-analysis of more than 2.3 million participants. Obes Rev.

[26] Angeli F, Reboldi G, Verdecchia P (2013). Hypertension around the world: new insights from developing countries. J Hypertens.

[27] Crane HM, Van Rompaey SE, Kitahata MM (2006). Antiretroviral medications associated with elevated blood pressure among patients receiving highly active antiretroviral therapy. AIDS.

[28] Kazooba P, Kasamba I, Mayanja BN, Lutaakome J, Namakoola I, Salome T (2017). Cardiometabolic risk among HIV-POSITIVE Ugandan adults: prevalence, predictors and effect of long-term antiretroviral therapy. Pan Afr Med J.

